# Infantile Cholestatic Jaundice: A Variant of Niemann-Pick Disease Type C2

**DOI:** 10.7759/cureus.69506

**Published:** 2024-09-16

**Authors:** Syed Mohammed, Ashikabanu Mujibur Rahman, Akshai R

**Affiliations:** 1 Paediatrics, Saveetha Medical College and Hospital, Saveetha Institute of Medical and Technical Sciences, Saveetha University, Chennai, IND

**Keywords:** autosomal recessive, hepatosplenomegaly, jaundice, niemann-pick type c2, npc1 and npc2 gene mutation

## Abstract

Niemann-Pick disease is an autosomal recessive lysosomal lipid storage disorder disease caused by mutations in either Niemann-Pick disease type C1 (NPC1) or the NPC2 gene. It has a wide range of symptoms that vary in severity, classified into three main types: A, B, and C1 and C2, based on genetics and the symptoms and signs. The usual presentation in the neonatal period is cholestatic jaundice, subsequently, it will develop hepatosplenomegaly in infancy, failure to thrive, ataxia, hypotonia, seizure, difficulty in speech, swallowing, and recurrent respiratory tract infection. In this case report, we describe the case of a five-month-old infant presenting with jaundice, developmental delay, and hepatosplenomegaly, and the diagnosis was confirmed by whole exome sequencing. The current treatment regimen includes frequent monitoring of liver function with symptomatic management like speech therapy, and nutritional therapy.

## Introduction

Niemann-Pick disease is an autosomal recessive disorder, caused by mutations in the SMPD1 gene for type A and B Niemann-Pick disease. Mutations in either NPC1 (95% of cases) or NPC2 (5% of cases) genes cause Niemann-Pick disease type C (NPC), resulting from deficiencies of sphingomyelin phosphodiesterase 1 gene expression. The disease has an overall prevalence is 1/7,700 live births, with a higher prevalence seen in the Ashkenazi population [1]. Most commonly occurring in the French population, the condition was first clinically described by pediatrician Albert Niemann in 1914 and later histologically by Ludwig Pick in 1927. The NPC1 and 2 genes encode proteins essential for regulating cellular cholesterol balance. NPC1 is a large transmembrane protein found in the lysosome's limiting membrane whereas NPC2 is a small, soluble lysosomal protein that binds to cholesterol within the lysosomal lumen. Deficiencies in this lysosomal protein disrupt intracellular lipid trafficking, leading to neurological impairment and death [2,3]. Children usually die because of infection or central nervous system manifestations.

Symptoms vary based on the type of Niemann-Pick disease and age of onset. In early infancy, there may be a delay in motor developmental milestones, while later stages might present with gait disturbances, poor academic performance, cataplexy in the late infancy and juvenile stages, psychiatric disturbances, and ataxia in adulthood. Other symptoms like dysphagia, dysarthria, dementia, and vertical supranuclear gaze palsy, with signs of cholestatic jaundice specifically in the neonatal period, and progress to hepatosplenomegaly in childhood [4]. This disorder is categorized into three types: A, B, and C. Type A begins in early infancy and is the most severe type and children with this type rarely live beyond 18 months of age. Type B is juvenile onset and lives a longer life compared to type A, but requires oxygen support for respiratory failure. Type C occurs early in life, with a child presenting with jaundice at or following birth, seizures, hypotonia, tremors, enlarged liver and spleen. Depending on the severity, type C children will die in childhood, while others live into adulthood.

When a child presents with hepatosplenomegaly, even if it is not a significant sign, the treating pediatrician should suspect this type of disease; this will prevent complications or further progression of the disease. Research done by Jiang et al. has shown that several circulating biomarkers are commonly used in clinical laboratories to detect Niemann-Pick disease, such as Cholestane-triol (C-triol), trihydroxycholanic acid (TCG), N-Palmitoyl-O-phosphocholineserine (PPCS). Of these, TCG is particularly advantageous due to its ease of sample handling, stability, and greater specificity in distinguishing between carriers and affected individuals. Therefore, the plasma TCG assay is recommended as the primary diagnostic tool and TCG is the only blood-based biomarker appropriate for newborn screening [5]. The diagnostic approach for Niemann-Pick disease involves genetic analysis. DNA testing of a blood sample can reveal specific gene mutations associated with Niemann-Pick disease types A, B, and C. An MRI of the brain may show loss of brain cells, but these changes might not be evident in the early stages of the disease. Treatment is only by supportive care.

## Case presentation

An infant, the firstborn of third-degree consanguineous parents, presents with yellowish discoloration of the eyes, abdominal distension, and delays in reaching age-appropriate milestones. Weighing 2.6 kg at birth, with an APGAR score of 8-9, she was delivered via cesarean section in a government hospital. The infant required admission to the neonatal intensive care unit for four days due to neonatal hyperbilirubinemia. At the time of birth, the child had no complaints except neonatal hyperbilirubinemia, for which phototherapy was given. Antenatally, the mother had no comorbidities like gestational diabetes mellitus, pregnancy-induced hypertension, epilepsy, etc. The mother had no history of exposure to radiation or any teratogenic drugs, and the pregnancy was uneventful. She had no history of abortion, stillbirth, or sibling death, and no known metabolic, degenerative, or chronic hereditary diseases or died unexpectedly in the family. The child had received immunizations appropriate for their age.

**Figure 1:**
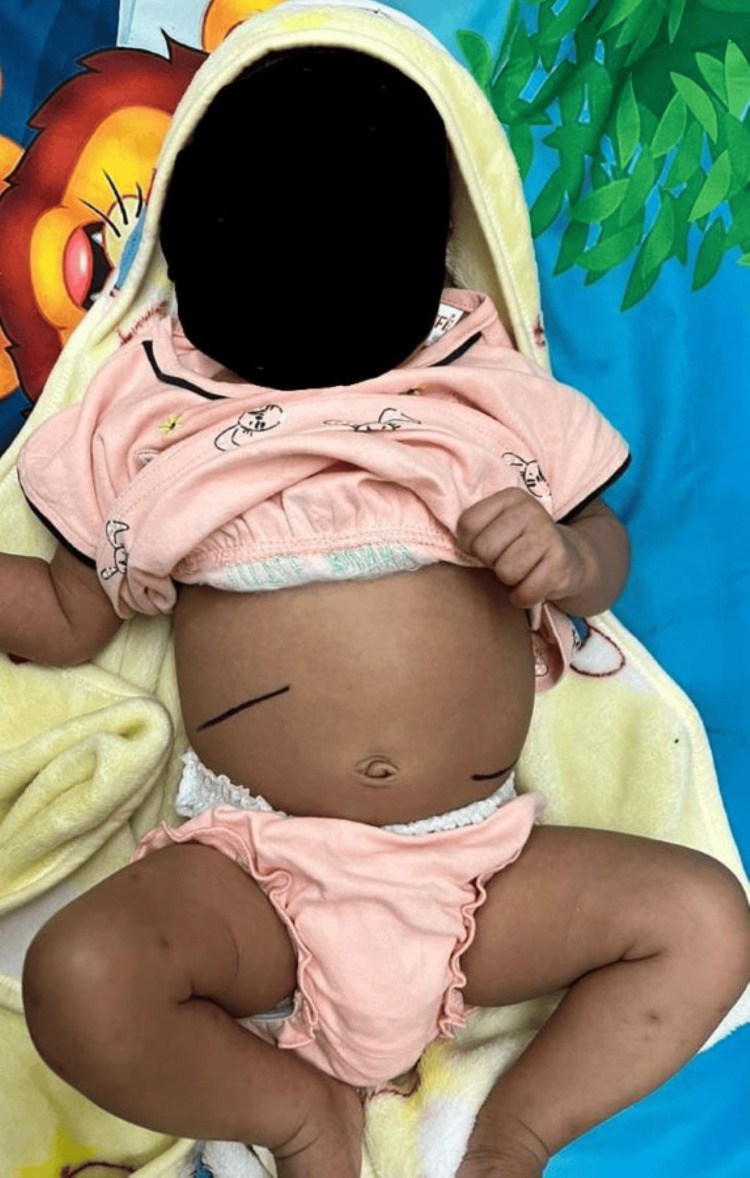
Infant with abdominal distension. Abdominal distension due to hepatosplenomegaly can be appreciated in the figure.

On examination, the infant was alert and active, with a weight of 4.8 kg, a length of 55 cm, a head circumference of 37 cm, and a mid-upper arm circumference of 11.5 cm, all falling above the -2 Z-score according to WHO growth charts. The anterior fontanelle was open and admitted the tip of a finger, while the posterior fontanelle was closed. Vitals and perfusion were within normal limits, and no visible facial dysmorphism was noted. The sclera and eyes were looking icteric. Per-abdomen examination showed hepatomegaly, with the liver palpable 3 cm below the right costal margin and the spleen palpable 6 to 7 cm below the left costal margin, near the umbilicus (Figure 1).

Investigations revealed a normal complete blood count, deranged liver function tests (Table 1), and nonreactive results for cytomegalovirus and rubella. The portal Doppler ultrasound showed an enlarged right lobe of the liver (7.2 cm) and an enlarged spleen (10.1 cm x 6.3 cm x 3.1 cm), leading to a diagnosis of hepatosplenomegaly.

**Table 1:** Liver function test.

Liver function test parameters	Result value	Reference range
Total bilirubin	2.90	0.2-1.2 mg/dL
Direct bilirubin	1.20	0.05-0.3 mg/dL
Indirect bilirubin	1.70	0.1-1 mg/dL
Serum glutamic oxaloacetic transaminase	120	20-67 U/L
Gamma glutamyl transferase	334	8-127 U/L
Globulin	1.70	2.0-3.5 g/dL
Albumin/Globulin ratio	2.06	1.0-2.0

The infant’s clinical presentation includes persistent hyperbilirubinemia, delayed developmental milestones, and hepatosplenomegaly characteristics of storage disorder. Gene analysis, specifically whole exome sequencing, suggests a mutation in the NPC2 gene, with exon 2-4 location of chromosome 14:g.deletion confirming a diagnosis of NPC2 (Table 2). Laboratory analysis and further imaging were consistent with this diagnosis. The child received supportive treatment for jaundice and later discharged with appropriate disease prognosis counseling. The patient keeps attending routine follow-up and has been given only supportive management.

**Table 2:** Clinical exome sequencing showing NPC2 gene mutation. Variants of uncertain significance related to the gene phenotype was detected. NPC, Niemann-Pick disease type C

Gene (Transcript)	location	variant	Zygosity	Disease (OMIM)	Inheritance	Classification
NPC2 (-)	Exons2-3	Chr14:g.del	Homozygous	Niemann-Pick disease type C2 (OMIM#607625)	Autosomal recessive	Likely pathogenic

## Discussion

Niemann-Pick disease is a rare, lipid storage disorder with an overall incidence rate of 1 in 120,000, in which types A and B affect 1 in 250,000 individuals, whereas Type C affects 1 in 150,000 individuals [6]. Isolated hepatomegaly and splenomegaly should always be the differential diagnosis of Niemann-Pick disease and an indication to investigate other symptoms [7]. Niemann-Pick disease is categorized into three types; type A is the most severe form and begins in the early infancy period. Type B usually occurs in the juvenile period, with the most common symptoms including ataxia and peripheral neuropathy. Types A and B occur when the deficiency of enzymes needed to break sphingomyelin. Types C1 and C2 may appear early in life or develop in adolescence, and occur when the body is not able to break down cholesterol and other lipids. Symptoms may vary depending on the type and time of presentation, but vertical supranuclear gaze palsy is the hallmark and strongest indicator, seen in 66% of patients with Niemann-Pick disease [8]. The clinical presentation that our patient had does not correlate to the classical phenotype, which made the diagnosis very challenging [2,3]. Due to sphingomyelinase deficiency, macrophages cannot metabolize lipids, which will produce irreversible damage. The most prominent findings are delay in attaining age-appropriate milestones, loss of motor skills, and organomegaly, especially liver and spleen [9]. Our patient presented with abdominal distension, and hepatosplenomegaly, which is the most common presenting feature in type A or B and occurs in over 90% of cases [10]. It will predispose to organ failure, especially liver failure, which is the first cause of death. The second cause of death is mainly respiratory failure. Histopathological examination of bone marrow and biopsy of organs such as the liver and spleen show pseudo-Gaucher foam cells.

Diagnosing Niemann-Pick disease is difficult because the disorder is rare, has varied onset and symptoms, presents with early non-specific signs, and requires complex laboratory testing. Moreover, the use of molecular diagnostics such as next-generation sequencing and gene panels tailored to specific phenotypes has enhanced the identification of Niemann-Pick disease. Studies done by Vanier et al. have revealed that the prenatal diagnosis of Niemann-Pick disease is based on low levels of sphingomyelinase and beta-glucosidase activity in amniotic cells [11]. Diagnosis and termination of pregnancy were based only on sphingomyelinase activity. Other differential diagnosis should be kept in mind, especially Gaucher disease, Tay-Sachs disease, and Metachromatic leukodystrophy. As of now, Niemann-Pick disease is not curable. Management focuses on symptomatic relief through supportive measures and palliation, involving a multidisciplinary approach. This may include a neurologist for movement disorders and seizures, a psychiatrist for behavioral disturbances, an ophthalmologist for gaze palsy, speech, and language therapies, and genetic counseling for families to address recurrence risks and consider prenatal diagnosis for future pregnancies if desired. Currently, studies are going on for enzyme replacement therapy with recombinant human acid sphingomyelinase [12,13]. The only approved drug for the treatment of Niemann-Pick disease is miglustat. Its mechanism of action involves reducing neuronal glycosphingolipid accumulation, delaying the onset of neurological dysfunction, and prolonging survival [14]. Early diagnosis and prompt management are needed to prevent early mortality. The patient must be kept under follow-up for conditions that may prevent disease progression, as it is a progressive disease that often develops complications early. Complications can include hepatic failure, respiratory failure, seizures, coronary artery disease, and others. The prognosis depends on the timing of the initial presentation: if the disease affects early infancy, survival typically does not extend beyond five years of age. However, if it affects the patient after five years, they may live up to 20 years for type C [15].

## Conclusions

Niemann-Pick disease, particularly type C2, is a fatal, multisystem disorder characterized by gradual and chronic debilitation. It typically begins with neonatal jaundice, and organomegaly of the liver and spleen, followed by the onset of developmental delays and hypotonia in children. Whole exome sequencing is the preferred method for confirming the diagnosis. Early mortality can be prevented through timely diagnosis, management of life-threatening symptoms, personalized physiotherapy, and genetic counseling. Although there is no cure, treatments like physiotherapy and speech therapy can improve the patient’s quality of life. Ongoing research continues to explore genetic mechanisms and potential therapies. Prenatal diagnosis enables informed decisions and early management strategies. Early detection remains crucial in optimizing outcomes for patients with NPC2, highlighting the need for prompt intervention.
